# Long-acting injectable antipsychotics and long-term functionality in schizophrenia

**DOI:** 10.1192/j.eurpsy.2026.10522

**Published:** 2026-07-31

**Authors:** P. Ifteni, A. Teodorescu, P. Petric, A. Popa

**Affiliations:** Transilvania university of Brasov, Brasov, Romania

## Abstract

**Introduction:**

Functional recovery is recognised as the most important target in schizophrenia, complementing symptomatic remission and relapse prevention [1]. Achieving and sustaining functionality remains challenging, as only a proportion of patients reach long-term stability.

The pharmacological strategies that support sustained functionality in real-world clinical practice remain insufficiently described. Long-acting injectables (LAIs), plays a central role in relapse prevention and illness stabilisation. While LAIs are often recommended for patients with poor adherence [2], recent evidence suggests they may also contribute to improved real-life outcomes in broader patient populations [3]. Understanding treatment patterns in patients who achieve high levels of functionality may provide important insights into the determinants of recovery.

**Objectives:**

To describe demographic and clinical characteristics, as well as antipsychotic treatment patterns, in a cohort of patients with schizophrenia identified by psychiatrists as achieving sustained functionality.

**Methods:**

Twenty certified psychiatrists in Romania each reported on five of their most functional patients with schizophrenia, resulting in a cohort of 100 individuals (50 men, 50 women). Functionality was determined by psychiatrists’ clinical judgement, supported by rating scales such as the Global Assessment of Functioning and the Social and Occupational Functioning Assessment Scale. Demographic characteristics, illness course, and psychopharmacological treatments were recorded and analysed. Ethical approval was obtained in accordance with institutional requirements.

**Results:**

The cohort consisted of patients selected as the five most functionally stable cases from each participating psychiatrist. The mean age was 43.2 ± 8.1 years, the mean age at illness onset was 26.7 ± 5.9 years, and the mean illness duration was 16.5 ± 7.4 years. The mean duration of remission was 7.2 ± 4.4 years.

Long-acting injectable (LAI) antipsychotics were prescribed in 72% of cases, with aripiprazole LAI most frequently used (42%), followed by paliperidone LAI (14%) and risperidone LAI (13%).

Among oral agents, olanzapine (14%), aripiprazole (8%), and clozapine (8%) were most represented, with smaller proportions on quetiapine, risperidone, cariprazine, and amisulpride. Combination antipsychotic treatment was reported in 10 patients.

**Conclusions:**

This study suggests that functionality in schizophrenia is frequently associated with prolonged remission, stable illness trajectories, and widespread use of LAI antipsychotics.

Aripiprazole LAI was the most frequently reported treatment. Previous studies have described benefits of aripiprazole LAI regarding tolerability, relapse prevention, and quality of life outcomes [4]. These results appear in line with previous evidence, while also possibly reflecting prescribing patterns or clinical selection.

**Disclosure of Interest:**

None Declared

